# The Emergence of Mem-Emitters

**DOI:** 10.1021/acs.nanolett.4c04586

**Published:** 2024-12-06

**Authors:** Victor Lopez-Richard, Igor Ricardo Filgueira e Silva, Alessandra Ames, Frederico B. Sousa, Marcio Daldin Teodoro, Ingrid David Barcelos, Raphaela de Oliveira, Alisson R. Cadore

**Affiliations:** † Departamento de Física, Universidade Federal de São Carlos, 13565-905 São Carlos, São Paulo, Brazil; ‡ Brazilian Synchrotron Light Laboratory (LNLS), Brazilian Center for Research in Energy and Materials (CNPEM), Campinas, São Paulo 13083-100, Brazil; ¶ Brazilian Nanotechnology National Laboratory (LNNano), Brazilian Center for Research in Energy and Materials (CNPEM), Campinas, São Paulo 13083-100, Brazil

**Keywords:** Mem-emitters, hysteresis loops, optical memory effects, van der Waals heterostructures, two-dimensional semiconductors

## Abstract

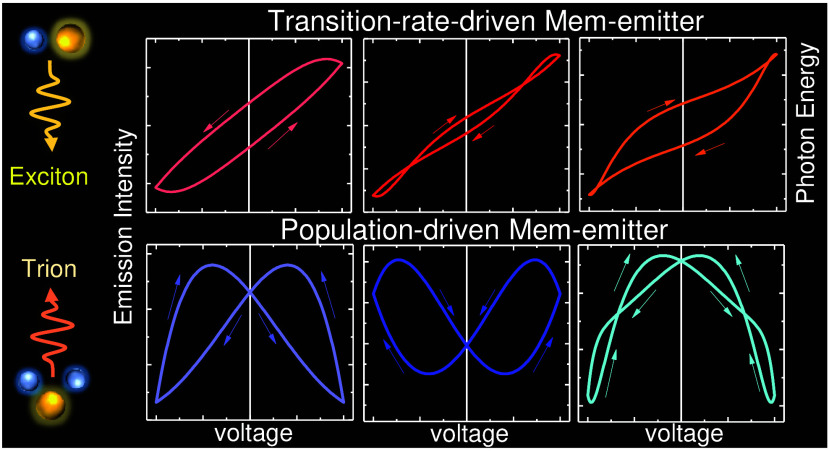

The advent of memristors and resistive switching has transformed solid-state physics, enabling advanced applications such as neuromorphic computing. Inspired by these developments, we introduce the concept of Mem-emitters, devices that manipulate light-emission properties of semiconductors to achieve memory functionalities. Mem-emitters, influenced by past exposure to stimuli, offer a new approach to optoelectronic computing with potential for enhanced speed, efficiency, and integration. This study explores the unique properties of transition-metal dichalcogenide-based heterostructures as a promising platform for Mem-emitter functionalities because of their atomic-scale thickness, tunable electronic properties, and strong light–matter interaction. When distinguishing between population-driven and transition rate-driven Mem-emitters, we highlight their potential for various applications, including optoelectronic switches, variable light sources, and advanced communication systems. Understanding these mechanisms paves the way for innovative technologies in memory and computation, providing insights into the intrinsic dynamics of complex systems.

In recent decades, the field of solid-state physics has been revolutionized by the emergence of memristors and the phenomenon of resistive switching.^1^ First theorized in the 1970s,^2^ memristors are circuit elements whose resistance can be altered based on their history. The underlying mechanism for resistive switching involves changes in the conductance of a material in response to electrical or other stimuli that interfere with internal state variables.^3^ This capability to modulate resistance has opened up exciting possibilities for neuromorphic computing^4^ and a variety of functionalities.^5−8^

Inspired by the concept of resistive switching in memristors, we propose a paradigm shift in optoelectronics. This approach focuses on manipulating the light emission properties of semiconductors to achieve memory functionalities using methods such as electromagnetic field modulation and proximity effects. We will refer to these devices as Mem-emitters where past exposure to light or other stimuli controls the characteristics of their emitted light. This enables a memory scheme where distinct light emission patterns represent different information states.

Mem-emitters and memristors share common performance parameters, such as response speed and memory robustness, but they excel in different applications. Mem-emitters uniquely combine ultrafast, interference-free optical readout with wavelength multiplexing and photonic network integration, making them ideal for hybrid optical-electronic systems.^9,10^ In contrast, memristors offer compactness, low power consumption, and suitability for large-scale neuromorphic networks, emphasizing their robustness and scalability.^7,8^

The main observables in the optical response of the Mem-emitter are the emission intensity and its energy position. To better frame, qualify and quantify the intensity of light emission, we can reduce it to its fundamental components: the population of the initial state, *N*, and the optical transition rate, 1/τ. The intensity *Q* can be expressed as
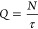
1In the simplest approximation, the optical transition rate can be expressed as
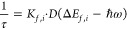
2where *K*_*f*,*i*_ is proportional to the elements of the optical transition matrix between the initial state (i) and the final state (f); and *D*(Δ*E*_*f*,*i*_ – ℏω) represents the energy conservation factor, which peaks when the photon energy (*ℏω*) approaches the energy difference between these two states (Δ*E*_*f*,*i*_), i.e, *ℏω* ≃Δ*E*_*f*,*i*_.

For the mem-emitting ability to emerge, the functions in eq 1 and 2 must depend not only on the external electromagnetic fields, F⃗, but also on a set of internal state variables, denoted as a vector of factors, x⃗. These internal state variables may include the density of activated or trapped carriers, internal electric polarization, redox reaction rates at the interfaces, filament formation rates, and other dynamic factors. Note that, in the presence of magnetic interactions, the spin degree of freedom must be considered. If the external fields vary, the time evolution of each emission intensity component: , , and  must be affected by dynamic relationships, that can be reduced in general terms to
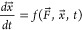
3This dependence ensures that the light emission intensity, *Q*, and energy position are influenced by both external fields and the internal state of the system, allowing for an adaptive emission behavior. Given that the optical emission rate of the Mem-emitter at any given time is influenced by the entire history of the external field evolution, its behavior is inherently non-Markovian. This characteristic allows Mem-emitters to be used in applications that require memory of past conditions, such as in neuromorphic computing and adaptive circuits. A key signature of this behavior is the appearance of stable hysteresis in either (or both) the emission intensity and energy position under periodic external driving, as depicted in Figure 1.

**Figure 1:**
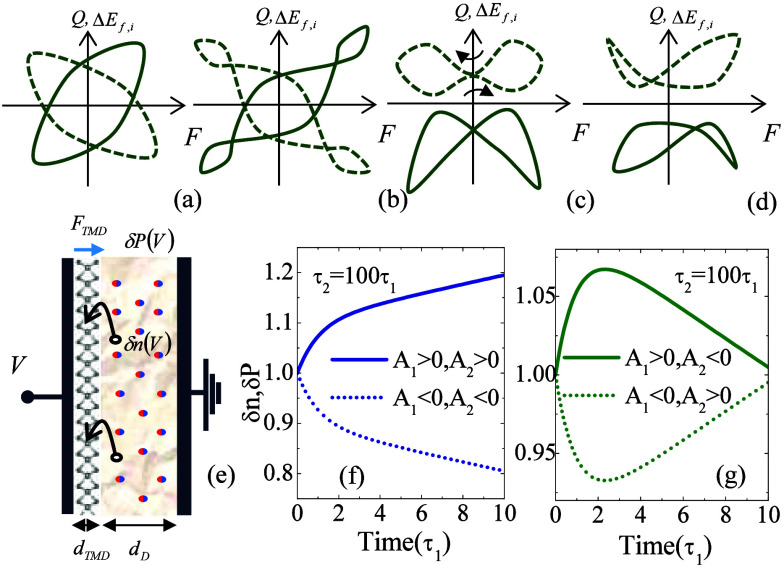
(a)–(d) Illustrative depiction of potential hysteresis loops of a Mem-emitter under periodic stimulus, showcasing various possible topological shapes: solid and dashed lines highlight contrasting loop configurations, and the arrows in panel (c) indicate a pinched, noncrossing loop. (e) Representation of a Mem-emitter consisting of a heterostructure based on a TMD monolayer of width *d*_*TMD*_ on its dielectric substrate of width *d*_*D*_, connected to two electrodes. Nonequilibrium carriers activated in the substrate and reaching the TMD layer are represented by *δn* along with the electric polarization fluctuation, *δP*, also occurring within the substrate. Time-resolved dynamics of either carrier or polarization fluctuations in the presence of two independent mechanisms with (f) monotonic and (g) nonmonotonic transients

The conditions under which these hysteresis loops appear and evolve by tuning external factors, along with their symmetry, and the number and positions of intersection points, are crucial elements for mapping the internal processes that generate them. According to the presence of an opening around the origin at *F* = 0, loops can be classified as (i) open loops [1 (a,b,d)], (ii) x-cross loops [1 (c) solid line], or (iii) pinched noncrossing loops [1 (c) dashed line]. On the basis of symmetry, loops can be categorized as (i) even-symmetric loops [1 (c)], (ii) odd-symmetric loops [1 (a,b)], or (iii) asymmetric loops [1 (d)]. Loops can also be classified according to the number of crossings: zero [1(a)], one [1(c) and 1(d)], two [1(b)], and more. Furthermore, the direction of the loop provides another classification criterion: (i) clockwise or (ii) counterclockwise loops. This classification helps to provide information about the internal dynamics of the systems. Specific cases will be discussed in the following.

Another aspect to be explored in the behavior of Mem-emitters is the contrast between population-driven and transition-rate-driven Mem-emitters. Population-driven Mem-emitters are characterized by their modulation through fluctuations in population density, *N*. In these devices, the primary mechanism influencing light emission is the variation in the number of charge carriers or emitters. This can be achieved through charge injection or extraction by adjusting the external electric field. The response time of these devices is typically governed by the rates of charge carrier injection and nonradiative recombination.^11^

On the other hand, transition rate-driven Mem-emitters are modulated primarily through the tuning of the optical transition rate (1/τ) with electromagnetic fields. In these devices, the focus is on altering the probability of radiative transitions between energy levels rather than the population density. The intensity of the emission is controlled by tuning the wave functions involved in the transition rate between states, which can be influenced by external electromagnetic fields and internal variables. The response time in transition-rate-driven Mem-emitters is influenced by the dynamics of the transition rates, which can be fine-tuned for specific applications. It is important to note that population and transition rate-driven mechanisms can coexist and might interfere in many systems and devices during the measurements.

Mem-emitters built on functional transion metal dychalcogenide (TMD) heterostructures and two-dimensional (2D) semiconductors, in general, present a promising platform for exploring Mem-emitter functionalities due to several properties.^12^ Their atomic-scale thickness allows for miniaturization of devices and results in a high surface-to-volume ratio, which enhances the interaction of the material with external fields and surrounding environments.^13,14^ Reduced dimensionality also facilitates better control over the electronic and optical properties of the material, making them highly sensitive to external stimuli such as proximity effects (of electrochemical nature)^15−17^ and high local electric fields.^18,19^ We should note that memory traces have already been observed in the optical response of devices based on TMDs,^20−22^ but have not yet been explored or characterized as the significant characteristic they evidently are.

Many TMDs, such as MoS_2_, WS_2_, and WSe_2_, exhibit a direct bandgap in their monolayer form, which is crucial for efficient light emission.^23,24^ The bandgap of these materials can be tuned by applying strain, electric fields, or by stacking different layers.^23,25^ This tunability is essential for developing transition-rate-driven Mem-emitters where controlling the emission wavelength and intensity is key. They also exhibit strong light-matter interaction due to their reduced dimensionality and high exciton binding energy.^23,26^ This results in enhanced optical absorption and emission efficiencies, making them ideal for optoelectronic applications.^27^ Furthermore, 2D TMDs possess well-resolved excitonic complex emission lines, such as neutral excitons and trions.^28−30^ These distinct emission lines offer a precise platform for probing mem-emitting abilities, as they provide clear, identifiable markers for studying the dynamics of light emission and its modulation under various external stimuli. Additionally, 2D semiconductors can be easily integrated with other materials, including traditional semiconductors, insulators, and metals, to form heterostructures and hybrid devices.^31,32^

To exemplify our Mem-emitter concept, we will use a device consisting of a 2D TMD monolayer placed on a dielectric substrate and connected to two electrodes, as show in Figure 1 (e). Note that this heterostructure also serves as the gate setup for a single-layer TMD transistor.^6,33,34^ In this capacitor configuration, under an applied bias, the electric field within the TMD monolayer, *F*_*TMD*_, is related to the applied voltage, *V*, and the built-in polarization in the substrate, *P*, by the following expression
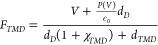
4where ϵ_0_ is the vacuum permittivity, *d*_*TMD*_ and *d*_*D*_ are the thicknesses of the TMD monolayer and the dielectric layer, respectively; χ_*TMD*_ is the dielectric susceptibility of the monolayer. Let us assume that the polarization of the substrate can be segmented into two contributions: one that varies instantaneously with the local field in the substrate region, *F*_*D*_, as *P*^(0)^ = χ_*D*_ϵ_0_*F*_*D*_, being χ_*D*_ the dielectric susceptibility of the substrate, and a fluctuation, *δP*, due to the leakage or generation of nonequilibrium dipoles. Thus, the total polarization can be expressed as
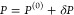
5

In that case, the local field at the TMD monolayer transforms to
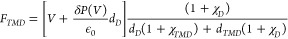
6which, under the condition *d*_*TMD*_ ≪ *d*_*D*_, simplifies to
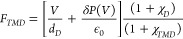
7Various concomitant carrier trapping processes can contribute to the polarization fluctuation, *δP* = *∑*_*j*_*δP*_*j*_. We will consider these processes to be independent, each with its own relaxation time, and each component *δP*_*j*_ will define an internal state variable, as described in eq 3 for our Mem-emitter definition. Under this approximation, the fluctuation for each process j can be described by^35^
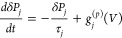
8where τ_*j*_ is the relaxation time and *g*_*j*_^(*p*)^(*V*) is the polarization transfer function dependent on the applied voltage *V* described in detail in ref (36). The sign of the transfer function determines whether the process is generative or depletive in nature. Furthermore, the trapping and release of carriers lead to fluctuations in the additional available charges, *δn* = *∑*_*i*_*δn*_*i*_, resulting from the independent generation of nonequilibrium channels. Each of these channels leads to charge fluctuations, *δn*_*i*_, adding new components to our internal state variable vector (eq 3), which, in analogy with eq 8, can be described using the relaxation time approximation
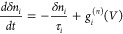
9where *g*_*i*_^(*n*)^(*V*) is the nonequilibrium carrier transfer function for each independent channel *i* that can be found defined in ref (3) in terms of microscopic elements. The most straightforward way to prove the coexistence of nonequilibrium processes with contrasting relaxation times is the analysis of the time-resolved transients of the observables under constant bias, *V*_0_. Under such conditions, using the charge fluctuation as a reference, the solution is
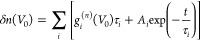
10with *A*_*i*_ = *δn*_*i*_(0) – *g*_*i*_^(*n*)^(*V*_0_)τ_*i*_, where *δn*_*i*_(0) is the initial condition when the *V*_0_ pulse is applied. The solution for the polarization fluctuation would be analogous. This equation highlights how different relaxation times manifest in the transient response of the system. Some results are illustrated in Figure 1 (f) and 1 (g). Figure 1 (f) shows monotonic trends, while Figure 1 (g) displays nonmonotonic trends for two concurrent processes with contrasting relaxation times differing by 2 orders of magnitude. The monotonicity is determined by the parameters *A*_*i*_, whose sign depends on the initial condition. Thus, no direct conclusion can be drawn on the character (sign) of the transfer functions, *g*_*i*_^(*n*)^(*V*_0_) or *g*_*j*_^(*p*)^(*V*_0_), in the case that the observables are related to either *δn* or *δP*, respectively.

Let us now discuss some particular cases of interest under periodic triangular voltage bias, *V*, with period T, as represented in Figure 2 (a). In this scenario, according to eq 8 and 9, the system response will evolve toward a periodic behavior once any transient effects, which depend on the initial conditions, have faded. Note also that any dynamic process, *k*, for which τ_*k*_ ≪ *T*, will manifest itself as instantaneous and its contribution to the fluctuation of either polarization or charge will be reduced to *δP*_*k*_(*V*) = τ_*k*_*g*_*k*_^(*p*)^(*V*) or *δn*_*k*_(*V*) = τ_*k*_*g*_*k*_^(*n*)^(*V*), respectively. In turn, under adequate voltage drives, the stable cyclic response can reveal signatures of memory effects through hysteresis in the observable versus voltage characteristics. According to eq 7, any hysteresis observed in the polarization versus *V* will be reflected in any observable that depends on the local electric field at the TMD monolayer.

**Figure 2:**
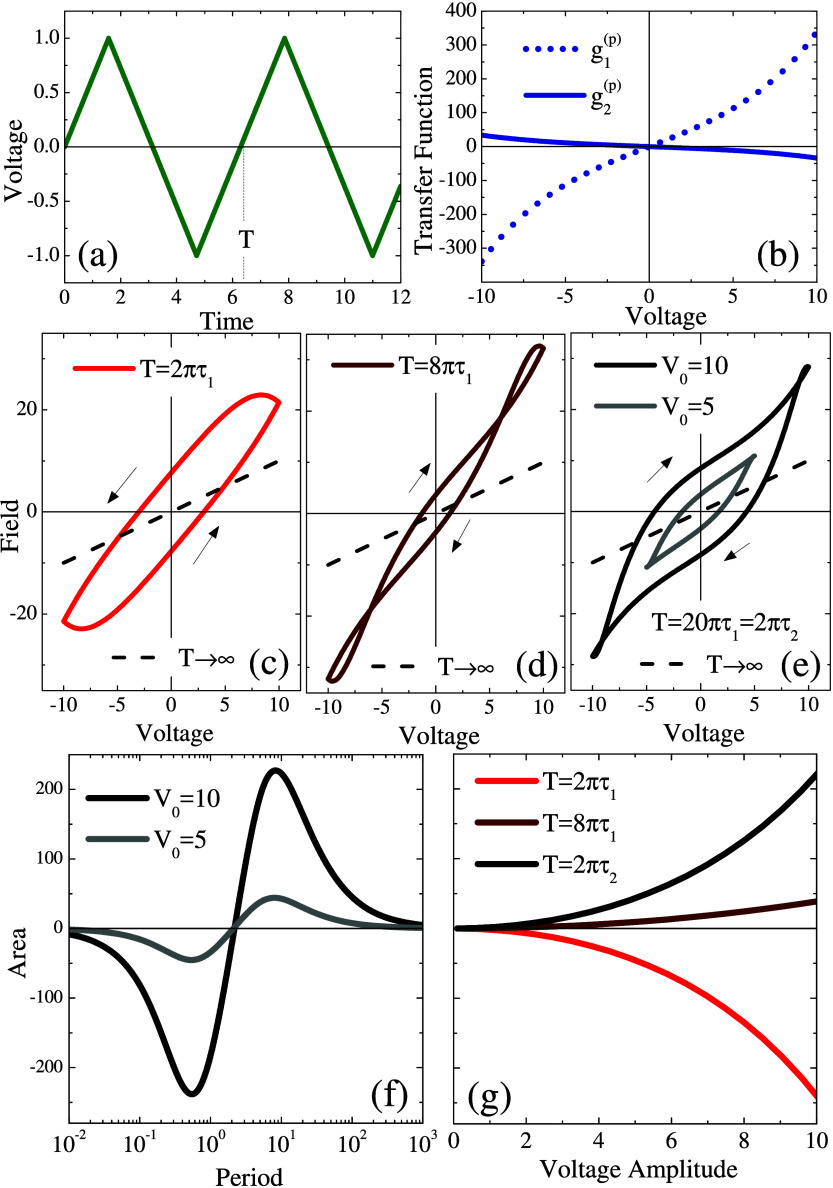
(a) Periodic voltage input. Electric polarization transfer function, *g*_1_^(*p*)^, that increases nonequilibrium polarization with voltage and, *g*_2_^(*p*)^, for a leaking channel. Stable cycles of the electric field at the TMD layer in the presence of the process activated by concomitant *g*_1_^(*p*)^ and *g*_2_^(*p*)^ for varying period: (c) *T* = 2*πτ*_1_, (d) *T* = 8*πτ*_1_, and (e) *T* = 20*πτ*_1_ = 2*πτ*_2_ (an electric field hysteresis for a voltage with half the amplitude has been added here). The arrows indicate the direction of the voltage sweep. (f) Loop area of the electric field as a function of the voltage period for two voltage amplitudes and (g) as a function of the voltage amplitude for various voltage periods. The parameters used in these calculations, not shown in the legends, can be found in the Supporting Information.

Two polarization transfer functions are illustrated in Figure 2(b). The function *g*_1_^(*p*)^ results in an absolute increase in polarization for both positive and negative bias, whereas *g*_1_^(*p*)^ characterizes a potential dipole leakage with an opposite sign relative to the bias. Assuming contrasting relaxation times for each of these processes, τ_1_ = τ_2_/10, and an increasing voltage sweeping period, the resulting local electric field hysteresis, according to eq 7 and 8, is shown in Figure 2 (c)-(e) (the field, *F*_*TMD*_, as a function of the electrode potential under stationary condition has been added as a reference). Note that the nature of generation or leakage determines the direction of the hysteresis loop: counterclockwise loops for delayed increased polarization and clockwise loops if prevailing leaking polarization mechanisms.^36−38^ In the cases where these processes interfere, the shape of the loop transforms into a multicrossing pattern, as depicted in Figure 2 (d). These loops collapse during very slow or very fast voltage sweeps, as depicted in Figure 2 (f) by the calculated loop area, *Area* = ∫_0_^*T*^*F*_*TMD*_*dV*, where a change in the sign indicates an inversion of the loop direction. Discussions on the conditions for maximum memory response can be found in refs (3, 39). The voltage amplitude also influences the area of the loops, as shown in Figure 2 (f).

Charge fluctuations can exhibit a wide variety of responses, as illustrated in Figure 3. Panel 3 (a) presents three symmetric, yet contrasting transfer functions that drive the charge fluctuations shown in panel 3 (b) under voltage cycles with *T* = 2*πτ*_1_, with τ_3_ = τ_1_ ≫ τ_2_. Under these conditions, *δn*_1_ and *δn*_3_ exhibit pinched hysteresis at *V* = 0 with opposite directions, while *δn*_2_ ≃ τ_2_*g*_2_^(*n*)^, following a quasi-stationary (instantaneous) response, without open hysteresis. As stated previously, it is possible to encounter concomitant processes with contrasting transfer functions and relaxation times. To illustrate this, we show the combined effects of adding *δn*_1_ + *δn*_2_, in panel 3 (c) and *δn*_1_ + *δn*_2_ + *δn*_3_ in panel 3 (d).

**Figure 3:**
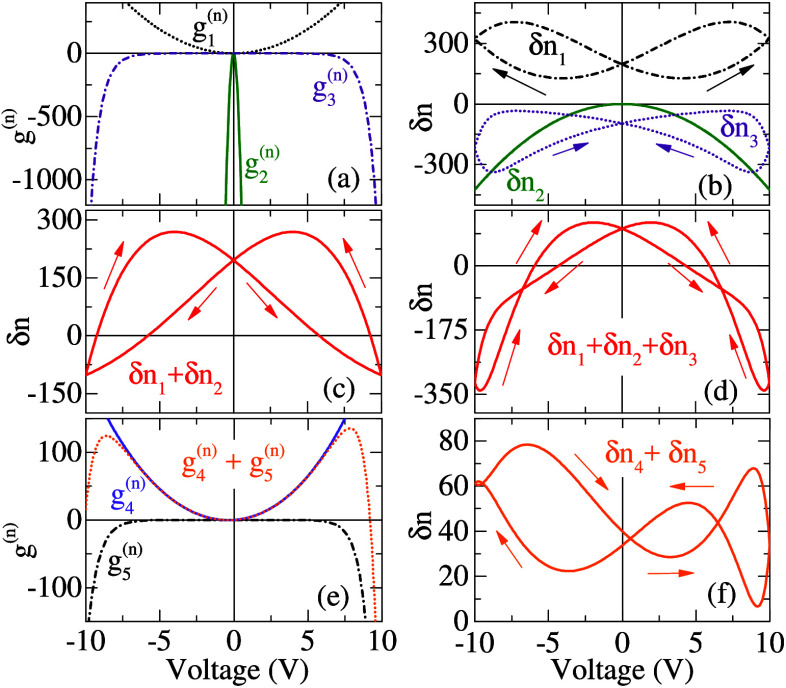
(a) Symmetric transffer functions for nonequilibrium charges of generation (positive) and trapping character (negative). (b) The corresponding loops for each of these three processes with τ_1_ = τ_3_ ≫ τ_2_ for voltage sweeps with period *T* = 2*πτ*_1_. (c) Combined effect of the *δn*_1_ + *δn*_2_ dynamics. (d) Combining *δn*_1_ + *δn*_2_ + *δn*_3_. (e) Two asymmetric transfer functions with contrasting characters and efficiencies and the combineed effect of them displayed in panel (f). The arrows indicate the orientation of the *V* sweep. The parameters used in these calculations, not shown in the legends, can be found in the Supporting Information.

The hysteresis crossing at *V* = 0 is disrupted when the inversion symmetry is broken. In Figure 3, we illustrate the combined effect of two transfer functions with contrasting characteristics. In this scenario, the asymmetry is controlled by a single parameter in the transfer function, as detailed in ref (3). The symmetry break alters the shape of the loop, as shown in Figure 3 (f), with the appearance of multiple loop intersections. These crossings can be attributed to the multiple sign changes of the function *g*_4_^(*n*)^+*g*_5_^(*n*)^ in panel 3 (e), although the positions of the intersections and the character change of the transfer functions (sign inversion with applied bias) are not correlated.

Note that in our proposed Mem-emitter architecture, all internal variables are contained within the substrate material, while all observables emerge from the TMD monolayer. These results underscore the pivotal role of the substrate’s chemical environment in modulating the local density of states^15,16,40−42^ and tuning the electronic structure, as demonstrated experimentally in ref (43). Now we must correlate the internal variables with the memory traces of the observables of our Mem-emitter: the emission intensity Q and the position of the emission line at *ℏω* = Δ*E*_*f*,*i*_ according to the approximations introduced in eq 1 and 2.

In devices with inversion symmetry break, such as the one proposed in Figure 1 (e), the modulation of the optical transition rate in the 2D TMD monolayer can be proportional to the local electric field^44,45^
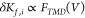
11This is also the case for the renormalization of the effective energy gap in excitonic complexes under inversion asymmetry conditions^18,44^
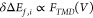
12We should also note that a perpendicular electric field can result in different slopes for the renormalizations of the exciton and trion energies, as outlined in ref (46).

Considering the optical transition rate (eq 2) 1/τ = *K*_*f*,*i*_(*F*_*TMD*_)·*D*[Δ*E*_*f*,*i*_(*F*_*TMD*_) – ℏω], we expect the modulation of the electric field memory with the internal variables to be translated into a transition-rate-driven Mem-emitter, following the trends reported in Figure 2, which could tune any excitonic complex emission. However, in the presence of electron fluctuation, *δn*, the intensity of trion emission, *Q*_*trion*_, has been shown to decouple from exciton emission, *Q*_*exc*_, given the expected ratio^47,48^
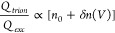
13If the effect of electron fluctuation prevails over electric field modulation, we can anticipate excitons to be prone to exhibit a transition rate-driven character in response to the local field, *F*_*TMD*_(*V*) as shown in Figure 2. In contrast, trions can demonstrate a mixed response, acting as population-driven Mem-emitters, according to the behavior of *δn*(*V*) depicted in Figure 3, while also showing transition rate-driven characteristics, similar to excitons. These predictions have been experimentally confirmed in ref (43), where a roadmap for the experimental realization of the presented devices is also described. Note that although a specific device serves as a tangible demonstration of underlying principles, examining its behavior in detail can elucidate broader Mem-emitter mechanisms and functionalities that extend to a wider range of systems, beyond those limited to 2D TMD architectures.

In summary, the concept of Mem-emitters introduces an approach to enhancing and diversifying optoelectronic device functionalities. In this perspective, understanding the differences between population-driven and transition-rate-driven Mem-emitters opens up a wide array of applications. Population-driven Mem-emitters offer the advantage of straightforward modulation of emitter density, making them suitable for optoelectronic switches, variable light sources, and applications where the control of active emitters is essential. In contrast, transition-rate-driven Mem-emitters provide refined control over emission properties, making them ideal for high-precision and advanced optoelectronic applications, such as communication systems and sensors that require precise emission control. By exploring these contrasting mechanisms and the information encoded in the light-emission patterns, it is possible to develop more versatile and efficient light-emitting devices tailored to specific needs in various technological fields. On the basis of this analysis, two conditions are sufficient for Mem-emitter abilities to emerge (or be resolved): (i) the proper tuning of external drives to match the internal time scale appropriate for a given internal state variable and (ii) the comparable strength of fluctuations of these variables with respect to their equilibrium or instantaneous reference values. Our findings suggest that the Mem-emitter functionalities are not limited to cryogenic conditions, as the internal state variable dynamics driving these effects can persist at elevated temperatures. Given the ubiquitous nature of the memory effects described, we can foresee that mem-emitting properties can also serve as a versatile tool for characterizing the intrinsic dynamics of complex systems beyond the functionalities of the Mem-emitter itself.

## Supporting Information Available

Detailed expressions for the transfer functions and tabulated parameters used to generate the results shown in Figures 2 and 3 (PDF)
