# Supplementary material for: The Emergence of Mem-Emitters
Source: Nano Lett. 2024 Dec 6;25(5):1816–22. doi: 10.1021/acs.nanolett.4c04586 (PMC11803706; doi:10.1021/acs.nanolett.4c04586)
Supplement: Supplementary file 1 — nl4c04586_si_001.pdf [file nl4c04586_si_001.pdf]

Supplementary Information file for: **The Emergence of Mem-Emitters**

Victor Lopez-Richard, Igor Ricardo Filgueira e Silva, Alessandra Ames, Frederico B. Sousa, Marcio Daldin Teodoro, Ingrid David Barcelos, Raphaela de Oliveira, and Alisson Ronieri Cadore

Parameters used to generate the results depicted in each figure of the manuscript.

The expression of the transfer function for the electric polarization fluctuation, according to Ref. [A] is

$$g_i^{(p)} = \frac{\lambda}{2}\left[exp\left(\frac{1}{2}\frac{\eta V}{k_B T}\right) - exp\left(-\frac{1}{2}\frac{\eta V}{k_B T}\right)\right]$$

The corresponding parameters used in Figure 2 were:

	λ	η	τ
$g_1^{(p)}$	100.0	10^{-2}	0.1
$g_2^{(p)}$	-10.0	10^{-2}	1.0

The expression of the transfer function for the fluctuation of nonequilibrium carriers, according to Ref. [B] is

$$g_i^{(n)} = \frac{\lambda}{\eta}\left[-2 + exp\left(-\frac{\alpha}{\alpha+1}\frac{\eta V}{k_B T}\right) + exp\left(\frac{1}{\alpha+1}\frac{\eta V}{k_B T}\right)\right]$$

In this case, a symmetry control factor, α, has been inserted and the corresponding parameters used in Figure 3 were:

	α	λ	η	τ
$g_1^{(n)}$	1.0	0.18	2×10^{-5}	1.0
$g_2^{(n)}$	1.0	100.0	2×10^{-5}	10^{-3}
$g_3^{(n)}$	1.0	10^{-6}	-10^{-3}	1.0
$g_4^{(n)}$	1.1	10^{-2}	10^{-4}	1.0
$g_5^{(n)}$	0.9	2×10^{-7}	-10^{-3}	1.0
$g_6^{(n)}$	1.1	-10^{-2}	10^{-4}	1.0
$g_7^{(n)}$	0.9	-2×10^{-7}	-10^{-3}	1.0

[A] Unveiling Ferroelectric-Like Behavior in Leaky Dielectrics: A Microscopic Model for Polarization Dynamics and Hysteresis Inversion. 2024; https://doi.org/10.48550/arXiv.2410.16084.

[B] Tuning the conductance topology in solids. Journal of Applied Physics 2023, 133, 134901; https://doi.org/10.1063/5.0142721
